# Effects of adult education on cognitive function and risk of dementia in older adults: a longitudinal analysis

**DOI:** 10.3389/fnagi.2023.1212623

**Published:** 2023-08-23

**Authors:** Hikaru Takeuchi, Ryuta Kawashima

**Affiliations:** ^1^Division of Developmental Cognitive Neuroscience, Institute of Development, Aging and Cancer, Tohoku University, Sendai, Japan; ^2^Smart Aging Research Center, Tohoku University, Sendai, Japan; ^3^Department of Advanced Brain Science, Institute of Development, Aging and Cancer, Tohoku University, Sendai, Japan

**Keywords:** adult education, dementia, cognitive functions, longitudinal, prospective observational study

## Abstract

**Introduction:**

Mid/late-life cognitive activities are associated with a lower rate of subsequent cognitive decline and lower subsequent dementia risk over time.

**Methods:**

In this study, we investigated the association between adult education class participation and subsequent cognitive decline and dementia risk over time after adjusting for baseline cognitive function and genetic risk of dementia, correcting for several potential confounding variables, using a large prospective cohort data of participants from the UK Biobank study followed from 2006 to 2010.

**Results:**

The results revealed that participation in adult education classes at baseline was associated with greater subsequent retention of fluid intelligence score. Cox proportional hazard models revealed that subjects who participated in adult education classes showed a significantly lower risk of incident dementia 5 years after baseline compared with those that did not at baseline.

**Discussion:**

In this study, we show that participation in adult education classes preceded greater retention of subsequent fluid intelligence and a lower risk of developing dementia after 5 years: this association did not change after adjusting for cognitive function at baseline or genetic predisposition to dementia. Accordingly, participation in such classes could reduce the risk of developing dementia.

## Introduction

In an aging society, the increase in dementia is a public concern. Extensive research has identified factors associated with a low risk of dementia. Cognitive activity is a factor associated with a lower risk of dementia. In fact, mid- to late- life cognitive activities are associated with a lower rate of subsequent cognitive decline (Vemuri et al., 2014). Furthermore, engaging in intellectual activities, defined as popular leisure activities requiring information seeking or processing, is associated with a lower incidence of dementia (Sajeev et al., 2016). Some studies suggest that there is a possibility of reverse causation, while others suggest otherwise. For example, recent large studies have shown that this association tended to disappear with longer follow-up and that the association was due to reverse causation (a preclinical tendency of dementia to result in lower intellectual activities) (Floud et al., 2021). However, this could be due to the waning effects of intellectual cognitive activities; for example, the effects of a shared environment (family environment that affects twin similarly) on intellectual abilities are stronger during childhood, tending to decrease later in life (Bouchard, 2009). A similar phenomenon may well be at play in this situation. Alternatively, other studies suggest that even after adjusting for baseline cognitive functions, engaging in cognitive activity in later adult life is associated with less subsequent cognitive decline (Vemuri et al., 2014).

Whether the correction of genetic predisposition [i.e., polygenic risk score (PRS)] toward dementia (Lourida et al., 2019), as well as cognitive abilities, affect the impact of adult education (a typical intellectual cognitive activity) on subsequent dementia risk over time and longitudinal cognitive changes has not been determined to date. Thus, we aimed to address this gap in the literature by using data on participation in adult education classes and relevant outcome measures from the UK Biobank.

We hypothesized that participation in adult education was associated with (a) a lower incidence of dementia and (b) greater retention of cognitive functions. This is based on previous research (Vemuri et al., 2014), showing that cognitive activity at baseline is associated with less subsequent cognitive decline even after adjusting for baseline cognitive function. These results show the crucial neuroprotective effects of adult intellectual activity, particularly when we consider the prevalence of dementia and cognitive decline in aging societies.

## Materials and methods

### Participants

This study used UK Biobank data, obtained from a prospective cohort study of a middle-aged population in the United Kingdom. The procedures have been described elsewhere (https://www.ukbiobank.ac.uk/media/gnkeyh2q/study-rationale.pdf). Briefly, participants visited one of 22 assessment centers across the UK for data collection. Baseline data were obtained for 502,505 participants. For this cohort, the study included data obtained from the first assessment visit (2006–2010) and those obtained from the third assessment visit (2014), which included the largest follow-up psychological test data. The following analyses were based on data from subjects, for whom all dependent and independent data were available. The descriptions in this subsection are largely reproduced from our previous study using the same methods (Takeuchi and Kawashima, 2021).

### Evaluation of adult education class

Participation in adult education classes was evaluated by the following question: “Which of the following do you attend once a week or more often? (You can select more than one)” (UK Biobank data file ID: 6160). Possible answers included “Adult education class,” items of other activities, “None of the above,” and “preferred not to answer.” In this study, subjects who did not answer this question and those who “preferred not to answer” this question were excluded. Among the rest of the subjects, subjects who selected “Adult education class” were considered to participate in adult education classes, and those who did not were considered to not participate in adult education classes.

### Sociodemographic and lifestyle measurements used as covariates

From the database, self-reported gender data (data field ID: 31) and age at the assessment visit (data field ID: 21003) were extracted. In addition, the neighborhood-level socioeconomic status at recruitment (cov1), education level at recruitment (cov2), household income (cov3), current employment status (cov4), body mass index (BMI) (cov5), metabolic equivalent of task hours (MET) (cov6), number of members in household (cov7), current tobacco smoking level (cov8), current alcohol drinking status (cov9), sleep length (cov10), depression score (cov11), diastolic blood pressure (cov12), and visuospatial memory performance [excluding lower two standard deviations (SD)] (cov13) were extracted from the database and included as common covariates across analyses, together with sex, age at baseline, PRS, and 10 genetic principal components supplied by the UK Biobank (data field ID: 22009). Genetic ethnic group UK Biobank data (data field ID: 22006) were split into Caucasoid or not and used as a stratifying or group factor. For additional details, refer to the Supplementary methods and explanations provided by the UK Biobank (data field ID: 22009). The descriptions in this subsection are largely reproduced from our study using the same methods (Takeuchi and Kawashima, 2021). Visuospatial memory performance was chosen as a covariate given that it is a complex cognitive performance available from most participants in the UK Biobank and involves memory function, which is relevant to dementia. We chose diastolic blood pressure, as it is more robustly associated with dementia risk over time in middle life compared with systolic blood pressure (Power et al., 2011).

### Cognitive measures

Cognitive measurements were performed at all visits. Briefly, tests were administered through a computerized touch-screen interface at each assessment center. This study used data on fluid intelligence, visuospatial memory performance, and reaction time. More details are provided in the Supplementary methods.

### Polygenic risk scores (PRS)

In this study, a PRS representing the genetic load of Alzheimer’s disease (AD) and dementia was calculated and used as a predisposition to genetic dementia, as previously reported in a representative study (Lourida et al., 2019). Herein, the PRS was calculated based on summary statistics from a meta-analysis of AD dementia in a European ancestry sample (Kunkle et al., 2019). For calculating the PRS, single-nucleotide polymorphisms (SNPs) were first selected for those associated with dementia with a threshold of *P* = 0.000001 in the studies mentioned above; then, standard quality control procedures were applied. Finally, 133 SNPs were selected. These criteria were selected according to a study that found that the PRS calculated from SNPs chosen based on this threshold were significantly associated with dementia risk (Moody et al., 2021). These 133 independent variants include the APOE region (defined as 44, 400–46, 500 kb on chromosome 19). In the above meta-analysis, the alleles associated with dementia in an individual were weighted based on the strength of their association with dementia, summed, and Z-standardized.

### Statistical analyses

Psychological data were analyzed using the Predictive Analysis Software, version 22.0.0 (SPSS Inc., Chicago, IL, USA; 2010). The descriptions in this subsection are reproduced from previous studies using the same methods (Takeuchi and Kawashima, 2021).

Analyses of covariance (ANCOVA) were used to investigate the associations between participation in adult education classes on the first assessment visit and changes in cognitive measurements from the first to the third assessment visits after correcting for confounding variables. Differences from the first to third assessment visits were calculated because the second assessment contained less psychological data than the third. The raw score changes (third assessment visit occasion data–first assessment visit occasion data) in (A) fluid intelligence, (B) visuospatial memory performance, and (C) reaction time was the dependent variables for each ANCOVA.

The independent variables included BMI level at the first assessment and genetic ethnicity as fixed factors, and sex, age at the first assessment visit, time (days) between the first and third assessment visits, cov1–13 values (except cov5: BMI level) at the first assessment visit, PRS of dementia, 10 genetic principal components, the score of the dependent variable of each analysis at the first assessment visit, and participation in adult education classes at the first assessment visit as covariates. We also added an interaction term between genetic ethnicity and PRS of dementia. We did not model the interaction between sex and the PRS of dementia, as the PRS is calculated for predicting dementia across sexes. Adding the interaction term between sex and PRS did not show significance or affect the main results.

Cox proportional hazard models were used to examine the relationships between participation in adult education classes at the first assessment visit and all-cause dementia. All-cause dementia was ascertained using hospital inpatient records and linkage to data from the death register. This method for determining dementia was used in representative UK Biobank studies (Lourida et al., 2019). For details, see the Supplementary methods. The following subjects were excluded from the analyses: (a) those already diagnosed with dementia at baseline, (b) those diagnosed with dementia or those who died within 5 years after baseline, (c) those with self-reported dementia or cognitive impairment at baseline, (d) those with self-reported dementia without a diagnosis in either hospital inpatient records or death register data, and (e) those with visuospatial memory performance <2SD. The time scale considered spanned from the time of the first assessment visit until around September 2021. Covariates were sex, age at the first assessment visit, cov1–13 values at the first assessment visit, PRS of dementia, 10 genetic principal components, score of the dependent variable at the first assessment visit, and participation in adult education classes at the first assessment visit. Additionally, the analysis was stratified based on genetic ethnicity. Participants who developed dementia within 5 years after baseline were excluded due to the possibility that the association between the variables of interest and subsequent incident dementia just reflects that dementia is already affecting behaviors before diagnosis or pre-clinical behaviors of dementia. This approach has been used in many studies of dementia and standard one (Bokenberger et al., 2014; Luojus et al., 2017; Armstrong et al., 2018; Deal et al., 2020; Pyun et al., 2021).

For psychological analyses, results with a *P* < 0.05 threshold were corrected for false-discovery rate (FDR) using the two-stage sharpened method (Benjamini et al., 2006) and were considered statistically significant. This correction was applied to the results of the four main analyses mentioned above and that did not include sensitivity analyses. In this FDR testing, for low *P*-values, the corrected *P*-values could be lower than the uncorrected ones. In these cases, the results were considered significant only when both corrected and uncorrected results had a *P* < 0.05, as previously suggested by Pike (2011).

## Results

### Basic baseline data

Basic baseline demographics and socioeconomic variable data for all participants at the first assessment visit are provided in Supplementary Table 1. Table 1 shows the baseline psychological variables of participants with and without dementia included in the analysis of dementia (Cox proportional hazard model). All simple correlation coefficients of the association between participation in adult education classes and psychological variables used in the following ANCOVAs were<0.15 in the baseline assessment of psychological analyses (categorical variables were treated as continuous variables in these calculations). These results excluded the possibility of multicollinearity in the following ANCOVA analyses. Additionally, supplemental logistic regression analyses including adult education class participation at the first assessment as a dependent variable and other covariates from the main analysis as covariates revealed that the PRS of dementia did not significantly correlate with adult education class participation at the first assessment (whole sample: *P* = 0.363, Caucasoid: *P* = 0.216, non-Caucasoid: *P* = 0.536), indicating that genetic predisposition toward dementia does not predict baseline adult education class participation among non-dementia participants.

**TABLE 1:** Baseline characteristics of participants with and without future incidence of dementia.

	No incident dementia (*n* = 279,448)	Incident dementia (*n* = 2,973)
	Mean	(SD)
Age	55.72 (8.05)	63.95 (4.76)
Townsend deprivation index	−1.48 (2.98)	−1.15 (3.2)
Education length	14.64 (5.02)	13.1 (5.25)
MET*	31.82 (35.28)	32.81 (38.54)
Diastolic BP	82.22 (10.12)	81.93 (10.13)
Visuospatial memory (errors)	3.68 (2.4)	4.39 (2.52)
Sleep length**	7.15 (1.02)	7.23 (1.21)
Depression score	5.55 (2.03)	5.68 (2.17)
Z score of PRS of dementia	−0.01 (1)	0.46 (1.2)
Fluid intelligence	6.25 (2.12) *N* = 96,785	5.65 (1.96) *N* = 858
Reaction time	548.4 (110.92) *N* = 278,719	602.16 (132.74) *N* = 2,948
	**Number**	**Percent**
Male number	133,235 (47.7%)	1,751 (58.9%)
**BMI**		
(a)Underweight (x ≤ 18.5)	1,295 (0.5%)	16 (0.5%)
(b) Normal (25 ≥ x > 18.5)	93,468 (33.4%)	875 (29.4%)
(c) Overweight (30 ≥ x > 25)	119,553 (42.8%)	1,265 (42.5%)
(d) Obesity (x > 30)	65,132 (23.3%)	817 (27.5%)
**Household income**		
(a) < £18,000	53,869 (19.3%)	1,237 (41.6%)
(b) £18,000–£30,999	68,847 (24.6%)	933 (31.4%)
(c) £31,000–£5, 1999	75,868 (27.1%)	501 (16.9%)
(d) £52,000–£100,000	63,305 (22.7%)	246 (8.3%)
(e) > £100,000	17,559 (6.3%)	56 (1.9%)
Currently employed	176,370 (63.1%)	737 (24.8%)
**Household number**		
(a) 1	51,208 (18.3%)	757 (25.5%)
(b) 2	126,529 (45.3%)	1,776 (59.7%)
(c) 3	44,515 (15.9%)	285 (9.6%)
(d) ≥ 4	57,196 (20.5%)	155 (5.2%)
Current alcohol intake	261,531 (93.6%)	2,654 (89.3%)
**Current smoking level (3 levels)**		
(a) No	251,327 (89.9%)	2,653 (89.2%)
(b) Only occasionally	7,826 (2.8%)	66 (2.2%)
(c) On most or all days	20,295 (7.3%)	254 (8.5%)
Genetic Ethnicity (non-Caucasoid)	41,782 (15%)	357 (12%)
Adult education class participation	20,844 (7.5%)	203 (6.8%)

*MET: Metabolic equivalent of task hours (MET). Physical activity level. **Sleep length: <3 h was converted to 3 h, and > 10 h was converted to 10 h. Details on measures are provided in the Supplementary methods.

### Longitudinal psychological analyses

**FIGURE 1:**
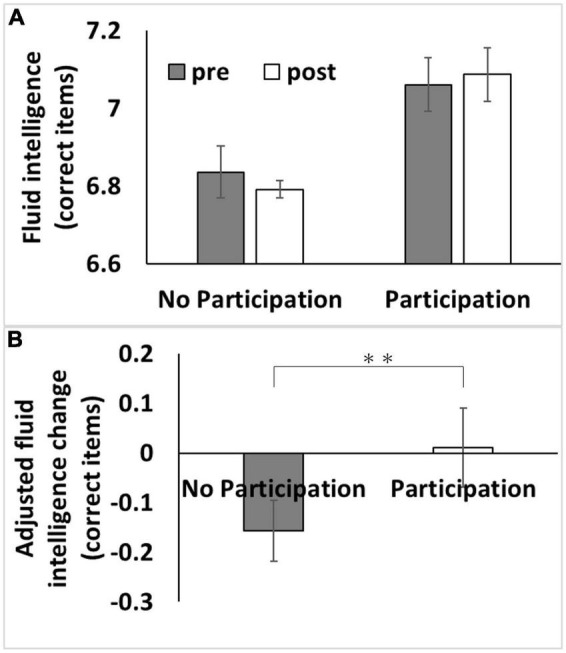
Association between baseline adult education class participation with subsequent changes in fluid intelligence. **(A)** Bars represent raw unadjusted pre- (1st assessment visit) and post-test (3rd assessment visit) measurements in each group and error bars represent the standard error of the mean. **(B)** Bars represent the adjusted values of changes in each group. After adjusting for confounding variables, including the baseline cognitive measure, an analysis of covariance revealed that baseline adult education class participation was associated with a relative greater longitudinal increase in fluid intelligence score [*P* < 0.05, corrected for false discovery rate (FDR)]. ^**^p < 0.01.

We used data from the first and third assessment visits for psychological data analyses. The mean age of the participants was 56.5 years [standard deviation (SD): 8.0, range: 37–73] at the first assessment, with a mean interval of 3,273.9 d (SD: 642.1, range: 1,400–5,043 d) for participants in both assessments. After correcting for confounding variables and multiple comparisons, an ANCOVA revealed that participating in adult education classes at baseline was associated with greater subsequent retention of fluid intelligence scores but not with reaction time or visuospatial memory performance (Figure 1 and Table 2).

**TABLE 2:** Association between participation in adult education classes and longitudinal changes in psychological measures (longitudinal ANCOVAs).

Dependent variables	Adult education		η 2	F	P (uncorrected)	P (FDR)
	Yes Adjusted changes (95% CI) *N*	No Adjusted changes (95% CI) *N*				
Fluid intelligence	0.011 (−0.146∼0.167) 829	−0.157 (−0.277∼–0.037) 9,323	6.70 × 10^–4^	8.681	0.003	0.005
Reaction time	55.69 (49.99∼61.38) 2,457	59.39 (54.90∼63.88) 27,290	9.30 × 10^–5^	3.552	0.059	0.041
Visuospatial memory (error)	0.117 (−0.056∼0.290) 2,438	0.178 (0.042∼0.315) 27,107	2.39 × 10^–5^	1.048	0.306	0.161
	***N***		**Exp (B) (95% CI)**	**Wald**	***p***	**P (FDR)**
Dementia after 5 years	282,421		0.813 (0703–0.940)	7.800	0.005	0.005

Adjusted changes indicate changes adjusted for covariates in ANCOVA tests.

The effect of adult education class participation in analyses using the whole sample (*P* = 0.003, η^2^ = 6.70 × 10^–4^) or using the Caucasoids’ sample (*P* = 0.001, η^2^ = 0.001) was significant, but not when using the non-Caucasoids’ sample (*P* = 0.687, η^2^ = 1.4 × 10^–4^). However, when the interaction term of ethnicity and adult education class was added to the main analysis, the effect of interaction between ethnicity and adult education class was insignificant (*P* = 0.111, *F* = 2.547).

### Prospective analysis of dementia

**FIGURE 2:**
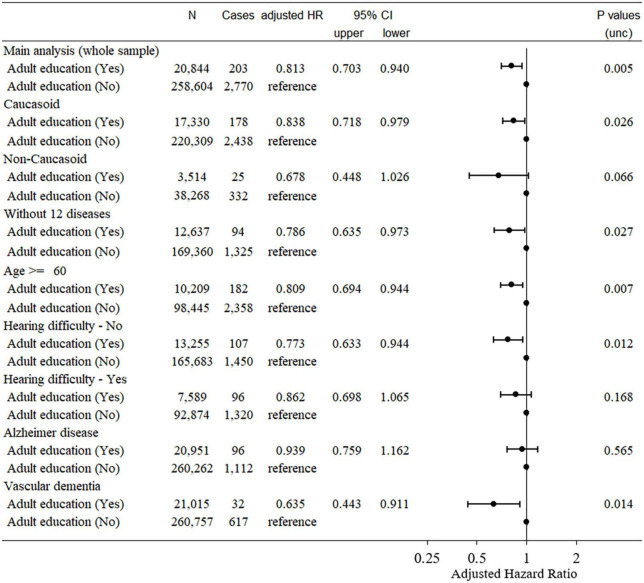
Statistical values and hazard ratios (95% CIs) for the associations between adult education class participation and incident dementia > 5 years after baseline in the UK Biobank data (main analyses and sensitivity analyses). Participants were categorized according to adult education class participation at baseline. 95% CI = 95% confidence interval; HR, hazard ratio.

Among the data from 502,505 participants in this study, 121 had self-reported dementia, 109 had records of diagnosed dementia before baseline, 750 participants had dementia records diagnosed within 5 years after baseline, and 8,462 died for other reasons during this period. Among the remaining participants, data from a total of 282,421 participants who had all effective relevant variables in the model were included in this analysis. Among these, 2,973 cases of dementia were observed. Cox proportional hazard models revealed that compared with subjects who participated in adult education classes had a significantly lower risk of incident dementia 5 years after baseline than those who did not (hazard ratio [HR]: 0.813, 95% confidence interval [CI]: 0.703–0.940, *P* = 0.005, Figure 2).

The HR of dementia in adult education class participants when only Caucasoids were analyzed (HR: 0.838, 95% CI: 0.718–0.979, *P* = 0.026) was comparable to that obtained only including non-Caucasoids (HR: 0.678, 95% CI: 0.448–1.026, *P* = 0.066) (the latter had a much smaller sample size).

### Sensitivity analyses of dementia

We then conducted a sensitivity analysis to observe the effects of participation in adult education classes among healthy subjects without comorbidities that could prohibit subjects from participating in adult education classes. Subjects were excluded due to major comorbidities such as diabetes, hyperlipidemia, angina, heart attack, high blood pressure, stroke, schizophrenia, depression, cancer, and other serious medical conditions/disabilities, see Supplementary methods for the determination of these comorbidities). The covariates and the statistical model were the same as in the main analysis.

In this sensitivity analysis, 183,416 participants (1,419 cases) were included. The Cox proportional hazard model subject who participated in adult education classes showed a significantly lower risk of incident dementia 5 years after baseline than those who did not (HR: 0.786, 95% CI: 0.635–0.973, *P* = 0.027). This suggests that the effect of adult education class participation on dementia risk over time is not due to the incapability of participants with major comorbidities to participate in such classes.

In addition, when analyses are limited to participants of ≥ 60 years at baseline, the effect of participation in adult education classes remained significant (HR: 0.809, 95% CI: 0.694–0.944, *P* = 0.007, Figure 2).

Since a recent study showed that speech-in-noise hearing impairment is related to a greater risk of dementia, we limited our analysis to subjects who had not reported such impairments at baseline. In this analysis of participants without speech-in-noise hearing impairments at baseline, participation in adult education was also associated with a significantly lower risk of incident dementia 5 years after baseline (HR: 0.773, 95% CI: 0.633–0.944, *P* = 0.012, Figure 2). A similar result was obtained when including only participants with speech-in-noise hearing impairment at baseline (HR: 0.862, 95% CI: 0.698–1.065, *P* = 0.168, Figure 2).

### Analysis of the type of dementia

Finally, we examined the relationship between types of dementia and adult education participation. In this analysis, as in the main analysis, patients diagnosed with dementia based on self-reports were excluded. For the analysis of AD, among subjects who had no medical record of AD or death before or within 5 years after baseline, we examined whether baseline participation in adult education classes was related to disease onset after 5 years using UK Biobank data (field ID 42020, 42021). Other procedures were the same as in the main analysis of all-cause dementia. Vascular dementia was similarly analyzed using UK Biobank data (field ID 42022, 42023).

Cox proportional hazard models revealed that subjects who participated in adult education classes did not show a significantly different risk of incident AD 5 years after baseline compared with those who did not (HR: 0.939, 95% CI: 0.759–1.162, *P* = 0.565, Figure 2). For vascular dementia, Cox proportional hazard models revealed that subjects who participated in adult education classes had a significantly lower risk of developing dementia 5 years after baseline than those who did not (HR: 0.635, 95% CI: 0.443–0.911, *P* = 0.014, Figure 2). Although, we did not statistically test whether the effects of adult education class differed for the two diseases, these results suggest that our findings and the main analysis of dementia are mainly driven by non-AD dementia subjects.

## Discussion

This study examined whether participation in adult education classes in middle to old age was associated with subsequent retention of cognitive function and a lower risk of developing dementia later in life, even after adjusting for genetic predisposition to dementia and baseline cognitive function. Our hypothesis was partially supported, as even after adjusting for the above factors, participation in adult education classes in middle to old age was associated with greater retention of fluid intelligence and a lower risk of developing dementia ≥ 5 years later. However, it did not appear to influence the retention of reaction time or visuospatial memory performance, implying that the observed association is domain-specific.

Even after adjusting for baseline cognitive function and genetic predisposition to dementia, subjects who participated in adult education classes had higher fluid intelligence retention than those who did not. This result is consistent with a study that showed an association between cognitive activity and longitudinal retention of greater cognitive functions, even after adjusting for baseline cognitive functions (Vemuri et al., 2014). The present study went beyond those results by newly demonstrating that genetic predisposition does not explain this association with dementia. However, despite the large sample size, no tendency for associations between adult education classes and memory performance was observed, indicating the specificity of the effects of participation in adult education classes. This may be due to the content of adult education classes, which may often be relevant to verbal cognition. This study used a visuospatial memory task that was speculative given the lack of information on the content of adult education classes. Future studies need to investigate this issue further.

In addition, even after adjusting for baseline cognitive function and genetic predisposition toward dementia, subjects who participated in adult education classes had a lower risk of dementia. This result is consistent with the abovementioned retention of higher-order cognitive functions in subjects with participation in adult education classes. As described in the Introduction, a previous study associated adult education class participation with the risk of dementia in the short to mid-term, but it was not significantly associated with the risk of dementia after 10 years (Floud et al., 2021). Based on this finding, it was suggested that the association reflects the preclinical condition of dementia, leading to less participation in adult education classes (Floud et al., 2021). However, although the effects of muscle or aerobic exercise wane once the training is stopped (e.g., Ismail et al., 2019), this does not mean that there was a lack of short-term effects of such training. Similarly, the effects of shared environmental factors on intellectual abilities are more significant during childhood but wane as one grows, but this does not imply that there were causal effects during childhood (Byrne et al., 2005). Additionally, the current findings show that the adjustment of baseline memory functions and genetic predisposition toward dementia do not alter these associations.

This study has a few limitations: first, this is an observational cohort study, not an interventional study. Although we corrected for a wide variety of confounding factors, baseline cognitive functions (Vemuri et al., 2014), and genetic predisposition to dementia, there may be some unspecified preclinical signs of dementia that we missed, which may be associated with participation in adult education classes at baseline. Ultimately, whether participation in adult education classes has a causal effect in preventing dementia risk and cognitive decline needs to be evaluated through randomized controlled trials. Second, the age range observed in this study was slightly skewed toward an earlier age of dementia onset, which may have decreased the statistical power of the analysis. Third, dementia diagnoses from healthcare records may lack sensitivity and there might be differences between the UK Biobank population and the general population. These are common limitations of UK Biobank’s prospective studies on dementia. Additionally, this study had no access to the specific content of adult education classes. In the future, it will be interesting to explore what type of adult education class participation is most associated with cognitive retention and reduced risk of dementia.

## Conclusion

In conclusion, the present study showed that participation in adult education classes was associated with higher retention of subsequent fluid intelligence and a lower risk of developing dementia ≥ 5 years. This association did not change after adjusting for cognitive function at baseline or genetic predisposition to dementia. Therefore, non-participation in these classes should be considered a risk factor for dementia. However, future interventional studies are required to fully demonstrate causality.

## Data availability statement

Publicly available datasets were analyzed in this study. The data is accessible upon the request to UK Biobank. Further inquires can be directed to the corresponding author.

## Ethics statement

The studies involving human participants were reviewed and approved by the North-West Multi-center Research Ethics Committee. The patients/participants provided their written informed consent to participate in this study.

## Author contributions

HT conceptualized the study, preprocessed, analyzed the data, and wrote the manuscript. RK played a key role in obtaining the relevant funding and supervised the study. Both authors read and agreed to the published version of the manuscript.

## References

[B1] Armstrong N. M. Carlson M. C. Schrack J. Xue Q.-L. Carnethon M. R. Rosano C. (2018). Late-life depressive symptoms as partial mediators in the associations between subclinical cardiovascular disease with onset of mild cognitive impairment and dementia. *Am. J. Geriatr. Psychiatry* 26 559–568. 10.1016/j.jagp.2017.11.004 29254675 PMC5940555

[B2] Benjamini Y. Krieger A. M. Yekutieli D. (2006). Adaptive linear step-up procedures that control the false discovery rate. *Biometrika* 93 491–507. 10.1093/biomet/93.3.491

[B3] Bokenberger K. Pedersen N. L. Gatz M. Dahl A. K. (2014). The type A behavior pattern and cardiovascular disease as predictors of dementia. *Health Psychol.* 33:1593. 10.1037/hea0000028 24364377 PMC4102675

[B4] Bouchard T. J. Jr. (2009). Genetic influence on human intelligence (Spearman’s g): How much? *Ann. Hum. Biol.* 36 527–544. 10.1080/03014460903103939 19634053

[B5] Byrne B. Wadsworth S. Corley R. Samuelsson S. Quain P. Defries J. C. (2005). Longitudinal twin study of early literacy development: Preschool and kindergarten phases. *Sci. Stud. Read.* 9 219–235. 10.1111/desc.12589 28812316 PMC5997458

[B6] Deal J. A. Power M. C. Palta P. Alonso A. Schneider A. L. Perryman K. (2020). Relationship of cigarette smoking and time of quitting with incident dementia and cognitive decline. *J. Am. Geriatr. Soc.* 68 337–345. 10.1111/jgs.16228 31675113 PMC7002272

[B7] Floud S. Balkwill A. Sweetland S. Brown A. Reus E. M. Hofman A. (2021). Cognitive and social activities and long-term dementia risk: The prospective UK Million Women Study. *The Lancet Public Health* 6 e116–e123. 10.1016/S2468-2667(20)30284-X 33516288 PMC7848753

[B8] Ismail A. D. Alkhayl F. F. A. Wilson J. Johnston L. Gill J. M. Gray S. R. (2019). The effect of short-duration resistance training on insulin sensitivity and muscle adaptations in overweight men. *Exp. Physiol.* 104 540–545. 10.1113/EP087435 30697876

[B9] Kunkle B. W. Grenier-Boley B. Sims R. Bis J. C. Damotte V. Naj A. C. (2019). Genetic meta-analysis of diagnosed Alzheimer’s disease identifies new risk loci and implicates Aβ, tau, immunity and lipid processing. *Nat. Genet.* 51 414–430. 10.1038/s41588-019-0358-2 30820047 PMC6463297

[B10] Lourida I. Hannon E. Littlejohns T. J. Langa K. M. Hyppönen E. Kuźma E. (2019). Association of lifestyle and genetic risk with incidence of dementia. *JAMA* 322 430–437. 10.1001/jama.2019.9879 31302669 PMC6628594

[B11] Luojus M. K. Lehto S. M. Tolmunen T. Brem A.-K. Lönnroos E. Kauhanen J. (2017). Self-reported sleep disturbance and incidence of dementia in ageing men. *J. Epidemiol. Community Health* 71 329–335. 10.1016/j.jalz.2014.08.104 28275046

[B12] Moody J. N. Valerio K. E. Hasselbach A. N. Prieto S. Logue M. W. Hayes S. M. (2021). Body mass index and polygenic risk for Alzheimer’s disease predict conversion to Alzheimer’s disease. *J. Gerontol. Ser. A* 76 1415–1422. 10.1093/gerona/glab117 33880516 PMC8277084

[B13] Pike N. (2011). Using false discovery rates for multiple comparisons in ecology and evolution. *Methods Ecol. Evol.* 2 278–282.

[B14] Power M. C. Weuve J. Gagne J. J. Mcqueen M. B. Viswanathan A. Blacker D. (2011). The association between blood pressure and incident Alzheimer Disease a systematic review and meta-analysis. *Epidemiology* 22 646–659. 10.7717/peerj.8189 21705906 PMC3640480

[B15] Pyun J.-M. Park Y. H. Lee K.-J. Kim S. Saykin A. J. Nho K. (2021). Predictability of polygenic risk score for progression to dementia and its interaction with APOE ε4 in mild cognitive impairment. *Transl. Neurodegener.* 10 1–9. 10.1186/s40035-021-00259-w 34465370 PMC8406896

[B16] Sajeev G. Weuve J. Jackson J. W. Vanderweele T. J. Bennett D. A. Grodstein F. (2016). Late-life cognitive activity and dementia: A systematic review and bias analysis. *Epidemiology* 27:732. 10.1097/EDE.0000000000000513 PMC5460628 27227783

[B17] Takeuchi H. Kawashima R. (2021). Diet and dementia: A prospective study. *Nutrients* 13:4500. 10.1097/EDE.0000000000000513 34960052 PMC8705494

[B18] Vemuri P. Lesnick T. G. Przybelski S. A. Machulda M. Knopman D. S. Mielke M. M. (2014). Association of lifetime intellectual enrichment with cognitive decline in the older population. *JAMA Neurol.* 71 1017–1024. 10.1001/jamaneurol.2014.963 25054282 PMC4266551

