# Supplementary material for: Effects of adult education on cognitive function and risk of dementia in older adults: a longitudinal analysis
Source: Front Aging Neurosci. 2023 Aug 23;15:1212623. doi: 10.3389/fnagi.2023.1212623 (PMC10480502; doi:10.3389/fnagi.2023.1212623)
Supplement: Supplementary file 1 [file Data_Sheet_1.docx]

**Supplementary online material**

**Supplemental Methods**

**Additional information on subjects**

Adults aged 40–69 years registered with the National Health Service (NHS) and living within 25 miles of the study's evaluation sites were invited by email to participate in the UK Biobank study. No exclusion criteria were applied for recruitment. This description is reproduced from our previous study using the same methods ([Takeuchi and Kawashima, 2021](#_ENREF_11)).

**Details on sociodemographic and lifestyle measures**

Descriptions regarding the covariates used in this study are mostly reproduced from our previous study using similar covariates ([Takeuchi and Kawashima, 2021](#_ENREF_11)).

(cov1) Neighborhood-level socioeconomic status was measured by the Townsend index of material deprivation ([Townsend, 1987](#_ENREF_13)). The status was calculated based on the subject’s home postcode and represents a composite index of four postcode-level socioeconomic status variables: household overcrowding, unemployment, non-home ownership, and non-car ownership). A higher score indicates a lower socioeconomic status. This value was obtained at recruitment and used for all analyses.

(cov2) Education level was based on self-reported data; the categories of participant choices were transformed into numerical values as described ([Okbay et al., 2016](#_ENREF_8)): “College or University degree” = 20 years; “A levels/AS levels or equivalent” = 13 years; “O levels/GCSEs or equivalent” = 10 years; “CSEs or equivalent” = 10 years; “NVQ or HND or HNC or equivalent” = 19 years; “Other professional qualifications, e.g., nursing, teaching” = 15 years; “None of the above” = 7 years; “Prefer not to answer” = missing. This value was obtained at recruitment and used for all analyses.

(cov3) Household income corresponded to the self-reported total income (before taxes) of the subject's household. The available choices were “<£18,000, £18,000–£30,999, £100,000, £31,000–£51,999, £52,000–£100,000, >£100,000,” “do not know,” and “prefer not to answer.” We converted these choices into ordinal variables between 1 to 5 (>£100,000 = 5) ([Shen et al., 2018](#_ENREF_10)) after excluding the answers “do not know” and “prefer not to answer,” as done elsewhere.

(cov4) Current employment status was used to describe the participants’ occupation. Possible responses to the variable were: “In paid employment or self-employed,” “Retired,” “Looking after home and/or family,” “Unable to work because of sickness or disability,” “Unemployed,” “Doing unpaid or voluntary work,” “Full or part-time student,” and “None of the above.” Multiple responses were allowed. Responses were either classified as “In paid employment or self-employed” or not.

(cov5) Body weight was measured using a Tanita BC-418 Body Composition Analyzer. Height was measured using a Seca height measurer. The body mass index (BMI) was calculated from the measured height and weight. BMI was treated as a categorical variable and separated into x > 30 (obesity), 30 ≥ x ≥ 25 (overweight), 25 ≥ x > 18.5 (normal), and x ≤ 18.5 (underweight), as typically done in the field.

(cov6) The physical activity level was calculated from the recorded items in the International Physical Activity Questionnaire short form and converted into a single measure of total physical activity in metabolic equivalent of task hours (MET). See the previous study for details ([Cullen et al., 2018](#_ENREF_3)).

(cov7) The number of people in their household (including institutions such as care homes) was self-reported. Answers were assigned one of four variables: 1 (single person), 2 (two people), 3 (three people), and 4 (four or more people), as done elsewhere ([Sarkar et al., 2008](#_ENREF_9)).

(cov8) Participants were asked about their current tobacco smoking status. Possible answers were 1 (No), 2 (Only occasionally), and 3 (Yes, on most or all days). “Prefer not to answer” responses were excluded.

(cov9) For current alcohol status information, study participants were asked to describe their current drinking status (never, previous, current, and prefer not to say). Then, individuals were classified as currently drinking (answer: “current”) or not (answer: “never,” or “previous”) after excluding “prefer not to say” responses.

(cov10) Sleep duration was assessed by the item “About how many hours sleep do you get every 24 h? (Please include naps)” Responses were coded as integers and used for analyses after counting sleep durations <3 h as 3 h and sleep duration >10 h as 10 h.

(cov11) Depressive symptoms were measured by the 4-item Patient Health Questionnaire-4 (PHQ-4) ([Batty et al., 2016](#_ENREF_1)), administered at all four patients’ visits to the assessment centers. This measurement has an area under the curve of 0.79 for its correlation with a depression diagnosis ([Khubchandani et al., 2016](#_ENREF_4)). For other information on the reliability and validity of this measurement technique, see ([Khubchandani et al., 2016](#_ENREF_4)).

(cov12) The diastolic blood pressure was measured using a digital BP monitor (Omron) or a manual sphygmomanometer if the digital monitor was unavailable (data-field IDs: 94, 4079). After performing 1–2 readings, the average was recorded as described previously ([Veldsman et al., 2020](#_ENREF_14)).

(cov13) Visuospatial memory was measured by the “pairs-matching” task. In this test, participants were asked to memorize the positions of six card pairs and match them from memory while making as few errors as possible. The score on the pairs-matching test corresponded to the number of errors made by each participant; therefore, higher scores reflected poorer cognitive functions.

**Details of cognitive measures**

We used data from tests administered to a large portion of subjects. The descriptions of these outcome measures were mostly reproduced from our previous study using similar covariates ([Takeuchi and Kawashima, 2021](#_ENREF_11)). Reasoning or fluid intelligence was evaluated using 13 verbal numerical logic and reasoning-type questions within a 2-min time limit and a maximum score of 13. The Cronbach alpha coefficient for each item was 0.62.

The reaction time was measured using a timed symbol matching test. In each trial, two cards with symbols were shown, and participants had to press a button as fast as possible when the two cards had matching symbols. The score was the mean response time across trials containing matching pairs. The Cronbach alpha coefficient for this task was 0.85.

Visuospatial memory performance was measured as described in the previous subsection.

**Polygenic risk score (PRS) calculation**

We followed the procedures of [Kunkle et al. (2019)](#_ENREF_5) and a published pipeline for calculating PRS in the UK Biobank ([Collister et al., 2022](#_ENREF_2)), as well as an accompanied homepage of the latter study (<https://2cjenn.github.io/PRS_Pipeline/>); the descriptions are mainly reproduced from these studies.

The results from a genome-wide association study of Alzheimer's disease based on the Stage 1 result of the meta-analysis of individuals of non-Hispanic white people ([Kunkle et al., 2019](#_ENREF_5)) were downloaded from <https://www.niagads.org/datasets/ng00075> and used as training dataset. As performed elsewhere, we used separate training and testing datasets to secure independence.

To select the single-nucleotide polymorphisms (SNPs) to be included in the PRS calculation, we first extracted all SNPs with *P* < 1.0 × 10^−6^ from the abovementioned meta-analysis according to previous research ([Moody et al., 2021](#_ENREF_7)). In UK Biobank cases with multi-allelic SNPs, we maintained the relevant alleles specified in the abovementioned meta-analysis. We used a hard-call-threshold of 0.1. We removed palindromic SNPs (i.e., SNPs where both alleles are (A, T) or (C, G)), with effect allele frequencies of 0.4–0.6 that could not be unambiguously distinguished between effect and noneffect alleles. We excluded SNPs with imputation information <0.4, excluded variants with minor allele frequency <0.005, and excluded those with a Hardy–Weinberg’s equilibrium of *P* < 0.000001.

We removed data from UK Biobank’s participants not used in a field (Field 22020), indicating that they did not meet their internal quality control checks for inclusion in the calculation of principal components. This quality control involves (a) missing rate in autosomes ≤0.02, (b) lack of outliers for missingness or heterozygosity, (c) not being in a maximal set of unrelated individuals, and (d) not being sex-discordant.

For the clumping procedure, we used PLINK 2 command --indep-pairwise (window size in variant counts: 1,000 kb, variant count for the window shift: 1, minimum r^2^ threshold to exclude SNPs: 0.2).

Rare variants causing rare familial diseases are not supposed to be included in the calculation of PRS due to the minor allele frequency cutoff value as are the cases of standard methods of calculation of PRS. Participants carrying rare familial mutations for dementia were included in the analysis, if not excluded based on exclusion criteria such as onset of dementia within 5 years after baseline.

For each participant in the UK Biobank, the number of associated alleles weighted by the beta, as reported by Kunkle et al., was counted, summed across all SNPs, and implemented with the --score command in PLINK2. The no-mean-imputation option was used to scale each individual’s score according to the number of non-missing SNPs without imputing missing SNPs.

**Ascertainment of dementia**

To ascertain all-cause dementia, we used previously established methods ([Lourida et al., 2019](#_ENREF_6)); the descriptions in this subsection are mostly reproduced from that study. Dementia was determined based on hospital inpatient records containing data on admissions and diagnoses from the Hospital Episode Statistics for England, Scottish Morbidity Record data for Scotland, and the Patient Episode Database for Wales. Additional cases were identified in death register data provided by the NHS Digital for England and Wales and the Information and Statistics Division for Scotland. Diagnoses were recorded using the International Classification of Diseases (ICD) coding system. Participants with dementia were identified as having a primary/secondary diagnosis (hospital records) or underlying/contributory cause of death (death register) using ICD-9 and ICD-10 codes for Alzheimer’s disease and other dementia classifications.

**Sensitivity analysis excluding subjects with comorbidities**

Next, we investigated the effects of participation in an adult education class on dementia risk over time change when removing subjects with major comorbidities that can preclude participation in the adult education class. The description in this subsection is mostly reproduced from our previous study ([Takeuchi and Kawashima, 2022](#_ENREF_12)).

In this analysis, we removed subjects with (1) self-reported doctor diagnosis of cancer (UK Biobank data field ID:2453), (2) self-reported doctor diagnosis of other serious medical condition/disability (UK Biobank data field ID:2473), (3) self-reported doctor diagnosis of diabetes (UK Biobank data field ID:2443), (4–8) doctor diagnosis of heart attack, stroke, high blood pressure, or angina (UK Biobank data field ID:6150), (9, 10) self-reported professional diagnosis of schizophrenia or depression (UK Biobank data field ID:20544), (11) doctor diagnosis of cancer (UK Biobank data field ID:2453), and (12) doctor diagnosis of other serious medical condition/disability (UK Biobank data field ID:2473). Finally, we re-ran the main analyses using the same covariates.

**Evaluation of speech-in-noise hearing impairments**

In the sensitivity analysis involving speech-in-noise impairments, we used data from the UK Biobank with data field ID: 2257, which asked subjects “Do you find it difficult to follow a conversation if there is background noise (such as TV, radio, children playing)?” and offered the responses: “yes,” “no,” “do not know,” and “prefer not to answer”. After excluding participants with responses of “do not know,” and “prefer not to answer”, participants were divided into those with responses of “yes” and those with responses of “no” and sensitivity analyses were conducted.

**References**

Batty, G.D., Mcintosh, A.M., Russ, T.C., Deary, I.J., and Gale, C.R. (2016). Psychological distress, neuroticism, and cause-specific mortality: early prospective evidence from UK Biobank. *J Epidemiol Community Health* 70**,** 1136-1139.

Collister, J.A., Liu, X., and Clifton, L. (2022). Calculating Polygenic Risk Scores (PRS) in UK Biobank: A Practical Guide for Epidemiologists. *Frontiers in Genetics* 13**,** 818574-818574.

Cullen, B., Newby, D., Lee, D., Lyall, D.M., Nevado-Holgado, A.J., Evans, J.J., Pell, J.P., Lovestone, S., and Cavanagh, J. (2018). Cross-sectional and longitudinal analyses of outdoor air pollution exposure and cognitive function in UK Biobank. *Scientific reports* 8**,** 1-14.

Khubchandani, J., Brey, R., Kotecki, J., Kleinfelder, J., and Anderson, J. (2016). The psychometric properties of PHQ-4 depression and anxiety screening scale among college students. *Archives of psychiatric nursing* 30**,** 457-462.

Kunkle, B.W., Grenier-Boley, B., Sims, R., Bis, J.C., Damotte, V., Naj, A.C., Boland, A., Vronskaya, M., Van Der Lee, S.J., and Amlie-Wolf, A. (2019). Genetic meta-analysis of diagnosed Alzheimer’s disease identifies new risk loci and implicates Aβ, tau, immunity and lipid processing. *Nature genetics* 51**,** 414-430.

Lourida, I., Hannon, E., Littlejohns, T.J., Langa, K.M., Hyppönen, E., Kuźma, E., and Llewellyn, D.J. (2019). Association of lifestyle and genetic risk with incidence of dementia. *Jama* 322**,** 430-437.

Moody, J.N., Valerio, K.E., Hasselbach, A.N., Prieto, S., Logue, M.W., Hayes, S.M., Hayes, J.P., and Initiative, A.S.D.N. (2021). Body mass index and polygenic risk for Alzheimer’s disease predict conversion to Alzheimer’s disease. *The Journals of Gerontology: Series A* 76**,** 1415-1422.

Okbay, A., Beauchamp, J.P., Fontana, M.A., Lee, J.J., Pers, T.H., Rietveld, C.A., Turley, P., Chen, G.B., Emilsson, V., Meddens, S.F., Oskarsson, S., Pickrell, J.K., Thom, K., Timshel, P., De Vlaming, R., Abdellaoui, A., Ahluwalia, T.S., Bacelis, J., Baumbach, C., Bjornsdottir, G., Brandsma, J.H., Pina Concas, M., Derringer, J., Furlotte, N.A., Galesloot, T.E., Girotto, G., Gupta, R., Hall, L.M., Harris, S.E., Hofer, E., Horikoshi, M., Huffman, J.E., Kaasik, K., Kalafati, I.P., Karlsson, R., Kong, A., Lahti, J., Van Der Lee, S.J., Deleeuw, C., Lind, P.A., Lindgren, K.O., Liu, T., Mangino, M., Marten, J., Mihailov, E., Miller, M.B., Van Der Most, P.J., Oldmeadow, C., Payton, A., Pervjakova, N., Peyrot, W.J., Qian, Y., Raitakari, O., Rueedi, R., Salvi, E., Schmidt, B., Schraut, K.E., Shi, J., Smith, A.V., Poot, R.A., St Pourcain, B., Teumer, A., Thorleifsson, G., Verweij, N., Vuckovic, D., Wellmann, J., Westra, H.J., Yang, J., Zhao, W., Zhu, Z., Alizadeh, B.Z., Amin, N., Bakshi, A., Baumeister, S.E., Biino, G., Bonnelykke, K., Boyle, P.A., Campbell, H., Cappuccio, F.P., Davies, G., De Neve, J.E., Deloukas, P., Demuth, I., Ding, J., Eibich, P., Eisele, L., Eklund, N., Evans, D.M., Faul, J.D., Feitosa, M.F., Forstner, A.J., Gandin, I., Gunnarsson, B., Halldorsson, B.V., Harris, T.B., Heath, A.C., Hocking, L.J., Holliday, E.G., Homuth, G., Horan, M.A., et al. (2016). Genome-wide association study identifies 74 loci associated with educational attainment. *Nature* 533**,** 539-542.

Sarkar, S.N., Huang, R.-Q., Logan, S.M., Yi, K.D., Dillon, G.H., and Simpkins, J.W. (2008). Estrogens directly potentiate neuronal L-type Ca2+ channels. *Proceedings of the National Academy of Sciences* 105**,** 15148-15153.

Shen, X., Cox, S.R., Adams, M.J., Howard, D.M., Lawrie, S.M., Ritchie, S.J., Bastin, M.E., Deary, I.J., Mcintosh, A.M., and Whalley, H.C. (2018). Resting-state connectivity and its association with cognitive performance, educational attainment, and household income in the UK Biobank. *Biological Psychiatry: Cognitive Neuroscience and Neuroimaging* 3**,** 878-886.

Takeuchi, H., and Kawashima, R. (2021). Diet and Dementia: A Prospective Study. *Nutrients* 13**,** Article 4500.

Takeuchi, H., and Kawashima, R. (2022). Effects of Body Mass Index on Brain Structures in the Elderly: Longitudinal Analyses. *Frontiers in endocrinology* 13**,** 824661.

Townsend, P. (1987). Deprivation. *Journal of social policy* 16**,** 125-146.

Veldsman, M., Kindalova, P., Husain, M., Kosmidis, I., and Nichols, T.E. (2020). Spatial distribution and cognitive impact of cerebrovascular risk-related white matter hyperintensities. *NeuroImage: Clinical* 28**,** 102405.

**Supplemental Table 1.** Baseline characteristics of UK Biobank participants included in this study (n = 502, 505)

| Item | No. (%) | Mean (SD) | Range |
| --- | --- | --- | --- |
| Sex |  |  |  |
| Female | 273,382 (54.4) |  |  |
| Male | 229,122 (45.6) |  |  |
| Missing | 1 |  |  |
| Age, years |  | 56.5 (8.0) | 37–73 |
| Missing | 1 (0.0) |  |  |
| Participation in adult education class |  |  |  |
| Yes | 36,226 (7.2) |  |  |
| No | 465,372 (92.6) |  |  |
| Missing | 910 (0.2) |  |  |
| Average total household income before tax |  |  |  |
| Less than £18,000 | 97,198 (19.3) |  |  |
| £18,000 to £30,999 | 108,177 (21.5) |  |  |
| £31,000 to £5,1999 | 110,772 (22.0) |  |  |
| £52,000 to £100,000 | 86,266 (17.2) |  |  |
| Greater than £100,000 | 22,929 (4.6) |  |  |
| Missing | 77,164 (15.4) |  |  |
| Townsend index of material deprivation |  | −1.3 (3.1) | −6–11 |
| Missing | 624 (0.1) |  |  |
| Employment status |  |  |  |
| In paid employment or self-employed | 287,149 (57.1) |  |  |
| Not in paid employment or self-employed | 212,404 (42.3) |  |  |
| Missing | 2,952 (0.6) |  |  |
| Highest education qualification (years) |  | 13.95 (5.1) | 7–20 |
| Fluid intelligence |  | 6.0 (2.2) | 0–13 |
| Missing | 339,748 (67.2) |  |  |
